# Genito-Urinary Surgery

**Published:** 1906-03-24

**Authors:** 


*(To be continued.)*

---

### GENITO-URINARY SURGERY.

**Movable Kidney.** — J. Hutchinson, Junr.,[1] in discussing this subject, first alludes to the views held by certain physicians upon the degree of importance to be attached to the condition. Goodhart, in 1891, declared that all subjects of movable kidney were of neuropathic disposition, and that it was foolish for surgeons to expect to cure the associated symptoms by operation. Sir James Sawyer has said that the diagnosis of movable kidney is only important because it enables you to assure the patient that she has a trivial complaint, and need take little notice of it. Many surgeons hold very different views. A condition which prevents active exercise, causes severe abdominal pain, and sometimes leads to serious changes in an important organ is in no sense an insignificant condition. In forty cases of nephrorrhaphy the author only once came across a kidney with a mesonephron. In 80 per cent. of the cases it is the right kidney alone which floats, in 10 per cent. the left only, and in 10 per cent. both organs. Once the author operated on a floating horse-shoe kidney, first fixing one side, with relief of symptoms on the corresponding side, and three years later operating on the other side. The predominance of the affection on the right side is attributed by the author to the different anatomical relations between colon and kidney on the two sides. On the left side the kidney is fixed by the firmly anchored splenic flexure and descending colon, on the right side the ascending colon in its lower part is on the inner side of the kidney, and the hepatic flexure does not anchor the kidney as the splenic flexure does on the left side. The writer then points out how the symptoms of incarceration or strangulation of a movable kidney may be caused by the rotation of the kidney in such a direction that its lower pole comes forwards, and the long axis of the organ, instead of being vertical, becomes horizontal, or nearly horizontal. This alteration of position may lead to temporary obstruction of the vessels of the kidney, or to kinking of the ureter, with temporary anuria. Owing to the nervous connections of the kidney, pain produced in it may be referred to various parts—for example, to the iliac crest or the leg. Also reflex vomiting may be a result of the sympathetic nerve irritation. The vertical or the forward displacement of the kidney may drag upon the biliary passages directly or upon the first part of the duodenum. Hutchinson has operated on six cases referred to him as cases of gall-stones or cholecystitis. A death has resulted from a floating kidney obstructing the duodenum and stomach by dragging upon a peritoneal band. Chronic gastric ulcer may be imitated in symptoms by movable kidney. The author recommends fixation of the kidney with four or five kangaroo tendons passed through capsule, cortex, and abdominal muscles. The operation fails to give relief in 20 or 30 per cent. of cases. Victor Bonney [2] prefers the term "injurious mobility" of the kidney to that of "pathological mobility," and asserts that the symptoms associated with the condition bear a striking relation to the presence of two signs. The first, and least important of the two, is the absence of "expiratory return"; the second is the rotation of the kidney on an antero-posterior axis, whereby the organ comes to lie obliquely or even transversely across the posterior parietes, with its hilum upwards. In examining patients for these signs, they must be placed in the standing posture, since the defect is caused by gravity; and, secondly, the use of the manœuvres termed "palpation nephro-leptique" by Glenard is recommended. The writer discusses the anatomical arrangements for the support of pedunculated organs in general in the body, and points out that no such organ is suspended by a neuro-vascular pedicle. There is always a device to prevent abnormal tension upon the nerves and vessels. The principal support of the kidney is the perinephric fascia which passes inwards from the kidney in two layers, one in front of, and the other behind, the neuro-vascular pedicle. This fascia is very strong to the inner side of the kidney, and blends with the fascia in front of and behind the aorta. The two physical signs mentioned above depend upon the relaxation of this fascia. The writer believes that an oblique or transverse position of a movable kidney is always a proof that there is injurious traction on the pedicle. In examining for this sign he advises that one hand, with the thumb in front, be used for compressing the loin, and that with the disengaged hand the kidney be passed backwards and forwards beneath the thumb of the compressing hand. The direction of the long axis of the kidney can thus be readily appreciated. It is the traction exerted by the kidney upon its neuro-vascular pedicle which causes the organ as it descends to rotate round the circumference of a circle of which the renal vessels are the radius. A kidney exhibiting this rotation always shows loss of expiratory return, but the latter may exist without the former. The author further states that he has had some half-dozen cases in which, with the symptoms of movable kidney, he found, not any descent of the organ, but only rotation into a transverse position immediately under the costal margin. The shortness of the renal pedicle probably accounts for the assumption of this position. In cases of backache in women this rotation should be carefully sought for as a possible cause of the trouble. Arnold Sturmdorf (New York),[3] writing on nephroptosis states that Wolkow and Delitzin demonstrated the functional passivity of all textural support of the kidneys and the dominating influence of intra-abdominal pressure. The correctness of this view is supported by the fact that when patients are placed with the upper part of the trunk elevated, and the abdominal cavity is opened, then kidney mobility will be revealed of the existence of which there was no evidence before the intra-abdominal pressure was diminished by the incision. Another contributory factor of great importance is the conformation of those depressed areas in the posterior abdominal wall normally occupied by the kidneys. Dislocated kidneys will be found associated with skeletal stigmata. The possessor of such a kidney probably had club foot or has acquired flat foot, shows a tendency to hernia, spinal curvatures, dislocations, fractures, and uterine and ovarian displacements. Nephroptosis has been found in infants. In the abdominal protrusion of rickets nephroptosis can usually be demonstrated. Kidney support is not a question of fat supply. Kidney dislocation once developed, nothing short of surgical fixation will re-establish its normal stability. H. M. W. Gray [4] says good results like those obtained by Treves and others with supporting instruments for movable kidney have not been realised in his experience. In any case there is a large number of patients with loose kidneys in which pad treatment cannot be borne. Hospital patients cannot, or will not, give the money and care necessary for pad treatment. The writer thinks failure to secure fixation by operation is often due to faulty technique, and, especially in Great Britain, to neglecting to remove the peri-renal fat-bearing tissue between the kidney and the posterior abdominal wall. The author moors the kidney with strips of gauze, which are removed at the end of seven or eight days.

[1] Clin. Jour., Oct. 25, 1905, p. 17. [2] Ibid., Oct. 18, 1905, p. 13. [3] Med. Rec., Jan. 13, 1906, p. 45. [4] Scot. Med. Jour., Jan., 1906, p. 35.

